# Draft Genome Sequence of an Antifungal Bacterium Isolated from the Breeding Environment of *Dorcus hopei binodulosus*

**DOI:** 10.1128/genomeA.00424-14

**Published:** 2014-05-15

**Authors:** Takehiko Kenzaka, Yasuhiro Yamada, Katsuji Tani

**Affiliations:** Environmental Science and Microbiology, Faculty of Pharmacy, Osaka Ohtani University, Tondabayashi, Osaka, Japan

## Abstract

*Burkholderia* sp. strain A1 was isolated from a decaying log present in the breeding environment of a stag beetle. The draft genome sequence indicates that strain A1 harbors many biosynthesis molecules, which have antimicrobial properties, and thus potentially eliminates the fungi by producing antifungal compounds, such as siderophores.

## GENOME ANNOUNCEMENT

The stag beetle genus *Dorcus* consists of 140 species, and the Japanese *Dorcus hopei binodulosus* is one of the largest types of stag beetle (1). Although the significance of gut microbes in the stag beetle gut has been recognized, other possible roles of these microbes have yet to be fully described (2–5). Adult *D. hopei binodulosus* species produce eggs in woody debris, and the larval stage consumes the decaying wood as nutrition. In artificial breeding environments, fungi are often present on the surface of the woody substrates and can inhibit the growth of the larvae. It has been known that the presence of *D. hopei binodulosus* eliminates fungi in breeding environments.

After the fungus *Trichoderma* was eliminated by the presence of *D. hopei binodulosus*, *Burkholderia* sp. strain A1 was isolated from the decaying log, which was used for egg laying in the breeding environment of the stag beetle. Strain A1 inhibited the growth of the fungus on Sabouraud agar. 16S rRNA sequence analysis revealed that strain A1 had 100% similarity to *Burkholderia gladioli* strain BgHL-01. Strain A1 was not detected in the breeding environments lacking *D. hopei binodulosus*. Therefore, the *Burkholderia* sp. strain A1 likely originated from the gut of *D. hopei binodulosus*.

The genome of strain A1 was sequenced by 100-bp paired-end sequencing on an Illumina Hiseq2000 sequencing system provided by the Hokkaido System Science Co., Ltd. (Sapporo, Hokkaido, Japan). The high-quality 23,110,275 reads were assembled *de novo* using CLC Genomics Workbench v. 6.5 (CLCbio, Cambridge, MA, USA), and the resulting contigs were curated by CodonCode Aligner v. 4.5 (CodonCode Corp., Centerville, MA, USA). The sequenced reads were mapped to the contigs again. About 99.6% of read bases were mapped to the updated contigs. The final assembly of the A1 genome produced 8,116,364 bp in 304 contigs, with an average length of 26,699 bp and a G+C content of 68.4%. The assembled contigs were analyzed in the RAST annotation server for functional annotation (6).

A total of 7,141 open reading frames (ORFs) were predicted, and 5,722 (80.1%) were functionally assigned. The genome contained 7,086 putative coding sequences (CDSs), 52 tRNAs, and 3 rRNAs. The presence of genes for the biosynthesis of secondary metabolites, such as nonribosomally synthesized antimicrobial peptides and polyketides, indicates the ability of stain A1 to inhibit the growth of fungi or other pathogens (7). Gene clusters for production of antimicrobial substances such as colicin V, syringomycin (8), gramicidin (9), arthrofactin (10), and siderophores (staphylobactin and pyoverdine) (11, 12) and many siderophore receptor/transporter genes were detected in the genome sequence. A multitude of genes related to antifungal activity indicate that strain A1 may create a more hospitable breeding environment for stag beetle larvae. We are currently exploring the commensalism of the antifungal bacterium as a means of preventing the suppressive action of fungi on stage beetle larvae.

### Nucleotide sequence accession number.

The draft genome sequence of *Burkholderia* sp. strain A1 has been deposited in the GenBank database with the accession number JJMR00000000.
